# Time-series modelling of dengue incidence in the Mekong Delta region of Viet Nam using remote sensing data

**DOI:** 10.5365/wpsar.2018.9.2.012

**Published:** 2020-03-21

**Authors:** Nga TT Pham, Cong T Nguyen, Maria Ruth B Pineda-Cortel

**Affiliations:** a Vietnam National Space Center, Vietnam Academy of Science and Technology.; b Department of Medical Technology, Faculty of Pharmacy, University of Santo Tomas.

## Abstract

**Objective:**

This study aims to enhance the capacity of dengue prediction by investigating the relationship of dengue incidence with climate and environmental factors in the Mekong Delta region (MDR) of Viet Nam by using remote sensing data.

**Methods:**

To produce monthly data sets for each province, we extracted and aggregated precipitation data from the Global Satellite Mapping of Precipitation project and land surface temperatures and normalized difference vegetation indexes from the Moderate Resolution Imaging Spectroradiometer satellite observations. Monthly data sets from 2000 to 2016 were used to construct autoregressive integrated moving average (ARIMA) models to predict dengue incidence for 12 provinces across the study region.

**Results:**

The final models were able to predict dengue incidence from January to December 2016 that concurred with the observation that dengue epidemics occur mostly in rainy seasons. As a result, the obtained model presents a good fit at a regional level with the correlation value of 0.65 between predicted and reported dengue cases; nevertheless, its performance declines at the subregional scale.

**Conclusion:**

We demonstrated the use of remote sensing data in time-series to develop a model of dengue incidence in the MDR of Viet Nam. Results indicated that this approach could be an effective method to predict regional dengue incidence and its trends.

According to the World Health Organization (WHO), (*1*) Viet Nam is among the top 10 countries with the highest reported number of dengue cases in the world (91 321 cases in 2012). Studies have shown that dengue epidemics in Viet Nam occurred cyclically every 3–5 years and peaked approximately every 10 years. (*2*) These cycles are thought to be influenced by the circulating viral serotypes, host immunity and climate oscillations. (*3*) Dengue transmission occurs throughout the year in Viet Nam with peak numbers of cases reported in the rainy season from May to October. (*4*) Since 2007, dengue has been recorded in 55 of the 63 provinces in Viet Nam, increasing from north to south with the Mekong Delta region (MDR) experiencing the highest incidence recorded during the years 2000 to 2016.

Several recent studies have aimed to better understand the dynamics of dengue and the influences of environmental factors on the disease and to better predict outbreaks. Climate factors, in addition to multiple human, biological and ecological determinants, influence the emergence and re-emergence of infectious diseases, including dengue, (*5*) which is transmitted by both the primary vector *Aedes aegypti* and the secondary vector *Aedes albopictus.* (*6*, *7*) Studies have found a significant correlation between rain and dengue incidence in Metropolitan Manila, Philippines from 1996 to 2005, (*8*) and a correlation between temperature, rain and dengue incidence in southern Thailand by multiple regression analysis. (*9*) On a regional scale, a review of the impacts of climate change on human health provided more evidence of the burden of climate change–attributable diseases and emphasized the uncertainty in attributing diseases to climate change, owing to a lack of long-term, high-quality data. (*10*) Climate change is likely to affect the seasonal and geographical distribution of dengue fever in the Asia–Pacific region, but more studies are needed that adjust for regional and subregional socio-environmental factors in the assessment of climate effects on dengue transmission. (*5*) Climate is only one of many environmental factors; changes in land cover by human settlements, the presence of water bodies, and vegetation type also affect dengue transmission processes. (*11*)

A range of approaches, including statistical modelling, mathematical modelling and spatial analysis, have been applied to demonstrate relationships between dengue and climate variables and to predict dengue cases and outbreaks. (*12*, *13*) Statistical models that are commonly constructed to predict dengue incidence cannot precisely predict the time and place of a dengue outbreak. However, they are able to quantitatively associate climactic factors such as rain, temperature and humidity with dengue epidemics at certain geographic areas with specific time lags. (*14*)

We assumed that there was a strong association between dengue incidences and climate variables; therefore, we applied a time-series autoregressive integrated moving average (ARIMA) model for dengue prediction in the MDR of Viet Nam. To minimize the limitations of climate data from meteorological stations in spatial and time scales, we used the most accessible remote sensing data for climate variables: the Global Satellite Mapping of Precipitation (GSMaP) for rain data and the Moderate Resolution Imaging Spectroradiometer (MODIS) land surface temperature _n/d_ (MOD11A2, US Geological Survey, Reston, VA, USA) for night (LST_n_) and day (LST_d_). Our aim was to investigate the capability of ARIMA to provide sufficient lead-time prediction of dengue (*15*) for a region of high incidence in a tropical climate. This was an effort to combine advanced geospatial data in a predictive model to assist public health control and response operations in the region.

## Methods

### Study site

**Figure 1:**
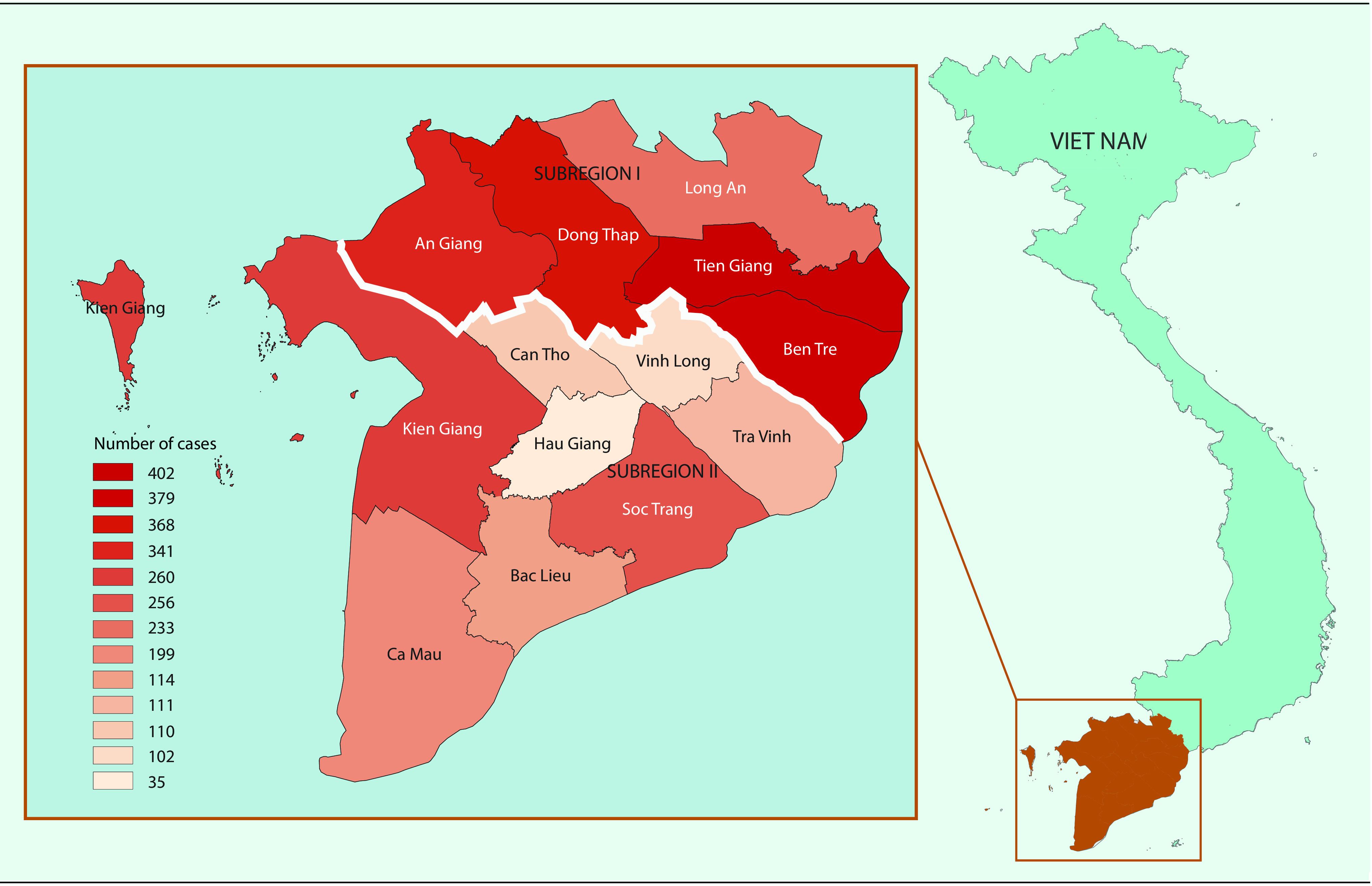
**Average monthly numbers of dengue cases for 13 provinces in the Mekong Delta region, divided into subregions I and II by a white line, 2000–2016**

The study was conducted in the lower MDR in south-western Viet Nam, using the average monthly number of dengue cases (**Fig. 1**). The MDR reported up to 65% of the total cases of dengue in Viet Nam during the period 2000 to 2016. This is a flat and low-lying area of 40 576 km^2^ covering 13 provinces within a complex network of rivers, channels and floodplains. We divided this region into two subregions: subregion I has the higher dengue incidence provinces (An Giang, Dong Thap, Long An, Tien Giang and Ben Tre) and subregion II has the lower incidence provinces (Vinh Long, Tra Vinh Can Tho, Hau giang, Soc Trang, Bac Lieu, Kien Giang and Ca Mau), separated by a white line in **Fig. 1**. Dengue fever has been recorded as the second most frequent reason for hospitalization among communicable diseases in this region. (*16*)

[**insert** Figure 1]

### Remote sensing data

**Figure 2:**
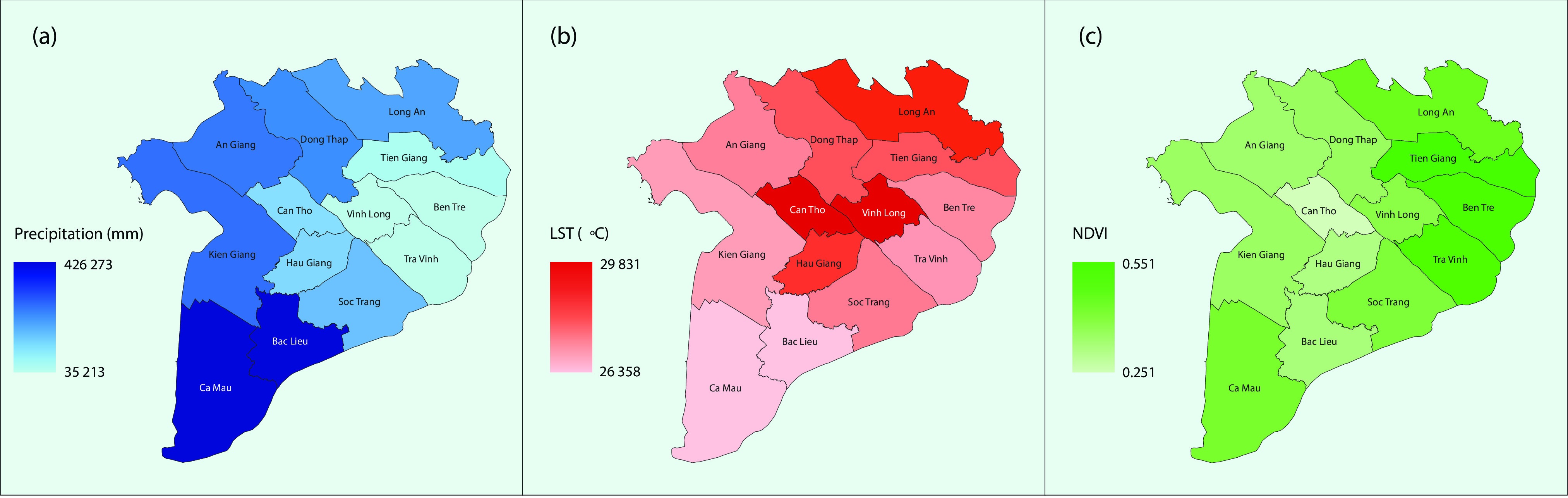
**Examples of monthly estimates for (a) GSMaP rain, (b) MODIS LST_d_ and (c) MODIS NDVI in the Mekong Delta region, November 2010**

We used GSMaP data as an alternative for surface rainfall measurement in an attempt to expand the use of remote sensing data with the advantages of spatial coverage with high resolution and temporal availability. The daily GSMaP-version 6 data (*17*) with a spatial resolution of 0.1 × 0.1 degrees were extracted and accumulated to calculate monthly rain. We used monthly land surface temperature data from MODIS LST_d_ and LST_n_ (MOD11A2) (*18*) with a 1 km spatial resolution as a proxy for air temperature. In addition to climate variables, one of the most commonly used remote sensing–derived environmental variables, the normalized difference vegetation index (NDVI) from MODIS (MOD13Q1) with a 250 m spatial resolution, was also used in the model for its influence on dengue. (*19*) These remote sensing–based parameters were aggregated to compute mean monthly variables for each province as examples presented in **Fig. 2**, showing clear spatial variations among provinces and between variables.

[**insert** Figure 2]

### Statistical analysis

We used the Box-Jenkins methodology (*20*) to fit ARIMA models to monthly dengue incidence in 12 provinces, using the statistical forecast package in RStudio software (version 1.1) (RStudio, Boston, MA, USA). (*21*) Dengue case definitions were based on WHO criteria (*22*) and collected through the disease surveillance systems according to Viet Nam’s Ministry of Health regulations. (*23*) The dengue cases reported from 2001 to 2015 were used for developing the time series model, and the cases during 2016 were used for validating the model. To avoid effects from the non-constant variance, we stabilized dengue counts by natural log transformation.

First, to confirm that ARIMA models were suitable for this analysis, we examined the data for seasonality and interannual variations of dengue incidence and climate and environmental variables (rain, LST_d_, LST_n_ and NDVI) during the period 2000–2016 for each province. Then, the adequacy of each model for each province was verified by histogram, by autocorrelation of the standardized residuals, and by the Ljung-Box test, similar to previous studies. (*24*-*26*) Next, the structure of the model followed the standard form for ARIMA, *(p,d,q)(P,D,Q)_s_*, where *p* is the order of autoregression; *d*, the degree of differencing; *q*, the order of the moving average; *P*, the seasonal autoregression; *D*, the degree of seasonal differences; *Q*, the seasonal moving average; and *s*, the seasonal period. Different ARIMA model forms (combinations of *p, d, q, P, D* and *Q*) were tested to fit the log-transformed time series data without environmental covariates. The best ARIMA model was selected as that with the lowest Akaike information criterion, (*24*) a measure of the relative goodness of fit of a model across the 12 provinces (Hau Giang province was excluded as it had politically separated from Can Tho province in 2004). Then, multivariate ARIMA models were fitted with log-transformed dengue cases in relation to all the climate variables with time lags that were chosen by their best correlation with dengue.

## Results

### Seasonal variation of dengue and climate  parameters

**Figure 3:**
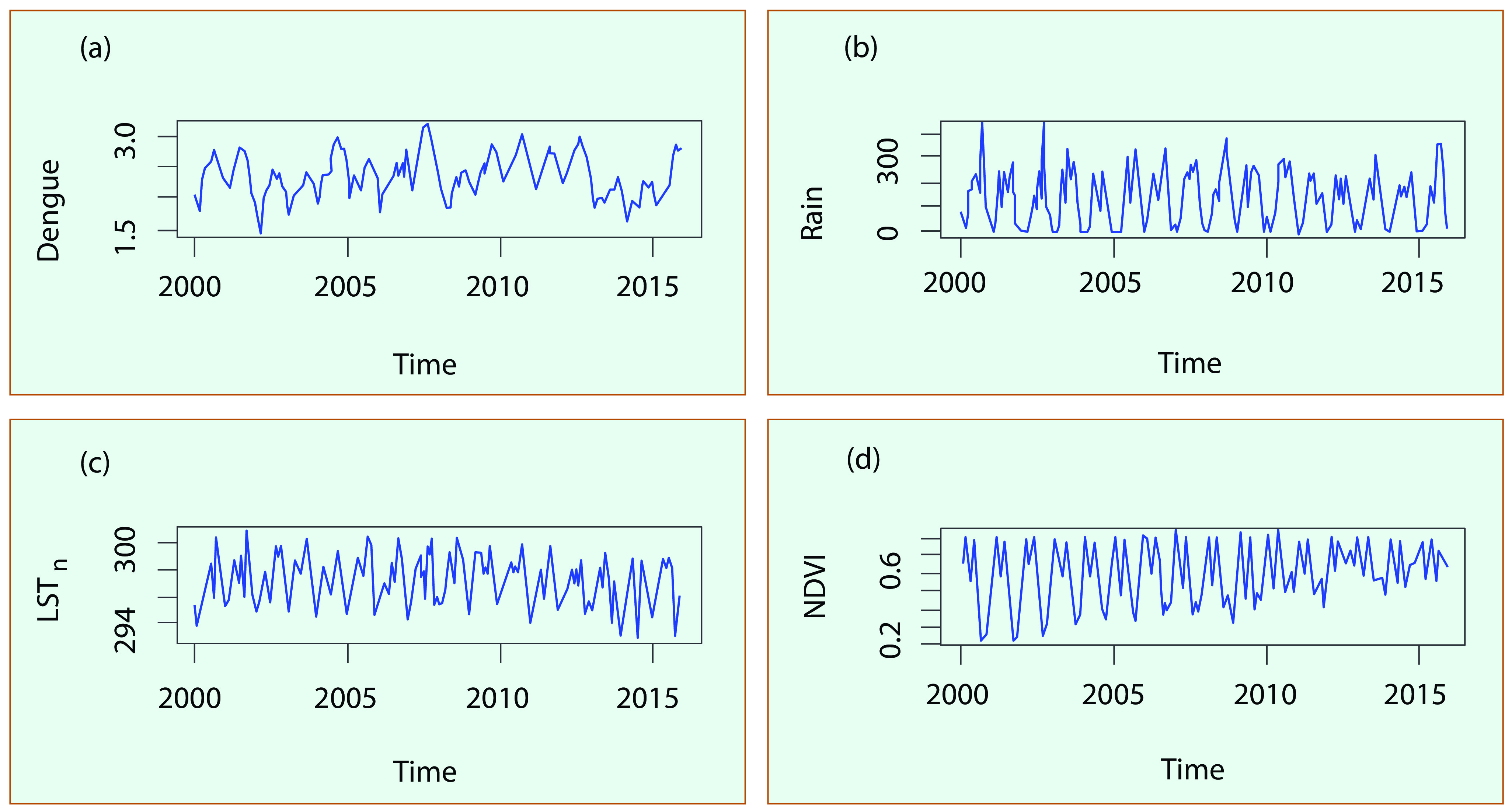
**Time series of (a) dengue, (b) rain, (c) LST_n_, and (d) NDVI for An Giang province, 2000–2015**

**Fig. 3** presents an example of the time series of dengue, rain, LST_n_ and NDVI during 2000- 2016 for An Giang province. The plots showed strong seasonal and interannual variations of all variables. We found this seasonal pattern apparent and consistent for all provinces in the region with higher dengue cases coinciding with rainy seasons. This enabled us to apply the ARIMA model to the entire MDR.

[**insert** Figure 3]

### Model in association with variables

**Fig. 4a** presents an example of an ARIMA fitting model plot for An Giang province and the comparison of fitted with reported dengue cases; **Fig. 4b** shows the regression function with its root mean square error value for the fitted period of 2000–2015. The final model for each province was confirmed by the Ljung-box test (*27*) of the residual with no correlation for fitted data.

**Figure 4a:**
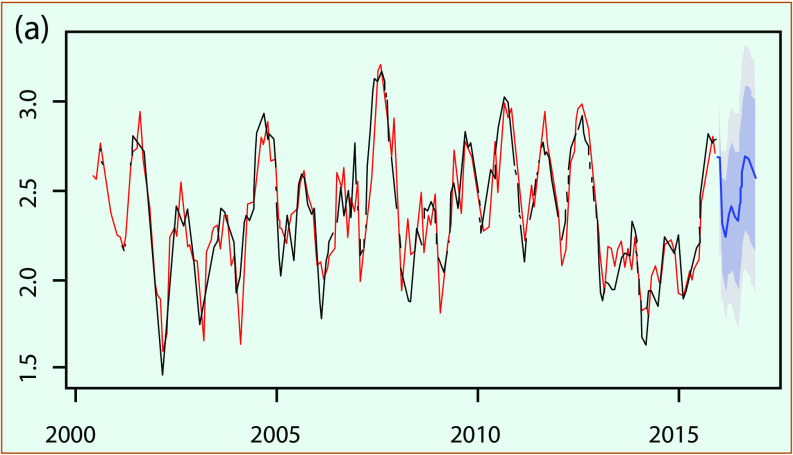
**Example of ARIMA model for An Giang province: (a) model fitting and (b) scatter plot of fitted and reported dengue cases**

[**insert** Figure 4a]

**Figure 4b:**
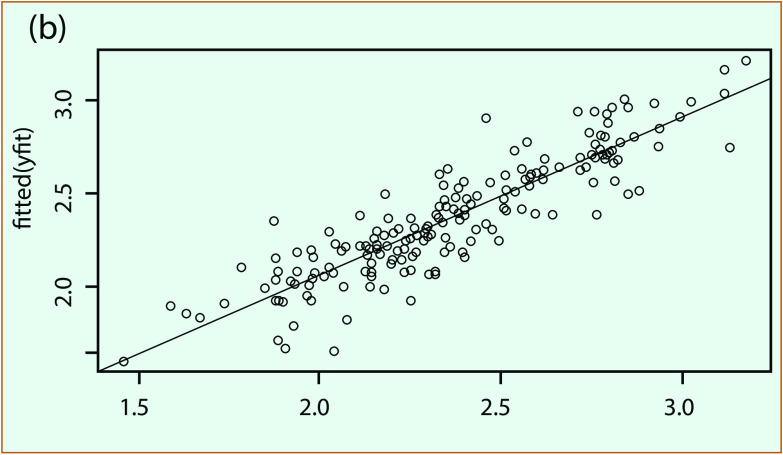
**Example of ARIMA model for An Giang province: (a) model fitting and (b) scatter plot of fitted and reported dengue cases**

[**insert** Figure 4b]

We generated 12 final models that closely fitted dengue incidence from every province in incorporating climate and environment variables as external predictors. After careful screening, those variables with the highest correlation with dengue incidence at specific time lags were kept in the final ARIMA models (**Table 1**) that show the correlation value, and the monthly time lag is shown in brackets. Correlations between these variables and dengue are spread over a range of time lags across provinces. Dengue had the strongest correlation with rain at a 1-month time lag across all provinces, reaching 0.60 at Long An province; dengue had a comparable correlation with LST_d_ and LST_n_ at 1-month to 4-month time lags. In addition, dengue had both positive and negative correlation with NDVI at 4-month to 5-month time lags. Notably, we found that the two subregions had different climate and environmental influences on dengue based on their correlations and time lags. In subregion I, dengue was found to have a higher correlation with rain with a 1-month time lag, and NDVI had a 5-month time lag; dengue was also associated with LST_d_ generally with a 4-month time lag and LST_n_ with a 1-month time lag. These findings are different than those for provinces in subregion II along the coast of the MDR where dengue was found to have a weaker correlation with rain and a negative correlation with NDVI with a 4-month time lag. For provinces in subregion II, dengue was correlated with a 1-month shorter time lag for LST_d_ but a 1-month longer time lag for LST_n_ compared to subregion I. For other provinces, including Vinh Long, Can Tho, and Kien Giang, dengue was found to have relatively low correlations with all variables and at mixed time lags. The variability in the association between dengue and climate and environmental factors across provinces in the study region emphasizes the need for a separate time-series model for each province.

**Table 1:** Final ARIMA models with correlation of dengue with climate and environmental variables for each  province in time lags

-	An Giang	Dong Thap	Long An	Tien Giang	Vinh Long	Bac Lieu	Ben Tre	Ca Mau	Can Tho	Tra Vinh	Kien Giang	Soc Trang
**r** with rain	0.515 (2)	0.509 (1)	0.604 (1)	0.471 (1)	0.342 (1)	0.291 (1)	0.536 (1)	0.437 (1)	0.482 (2)	0.370 (1)	0.403 (1)	0.314 (1)
**r** with LTSd	0.313 (4)	0.436 (4)	0.364 (4)	0.520 (4)	0.281 (4)	0.267 (3)	0.642 (3)	0.207 (3)	0.253 (4)	0.413 (3)	0.446 (2)	0.338 (2)
**r** with LSTn	0.444 (1)	0.522 (1)	0.394 (1)	0.339 (2)	0.296 (2)	0.354 (2)	0.501 (2)	0.304 (2)	0.388 (2)	0.449 (2)	0.410 (1)	0.461 (2)
**r** with NDVI	0.363 (5)	0.381 (5)	0.420 (5)	0.271 (5)	0.155 (5)	−0.402 (4)	−0.376 (4)	−0.392 (4)	0.190 (5)	−0.333 (4)	0.195 (4)	−0.297 (4)

r = correlation coefficient of dengue with difference variables

bracketed numbers = time lag

### Model validation

The best time-series ARIMA model with final independent variables found for each province was applied to predict dengue for the period from January to December 2016. In most cases, rain and LST_n_ remained in the final model; LST_d_ and NDVI were occasionally removed when correlations were less than 0.25. We compared predicted dengue with reported cases for the whole MDR, as shown in **Fig. 5**. The results showed that predicted dengue in every province closely followed the trend of reported data (**Fig. 5a**) and that these data are in good linear regression with the square of correlation of 0.65 for the whole region (**Fig. 5b**).

**Figure 5a:**
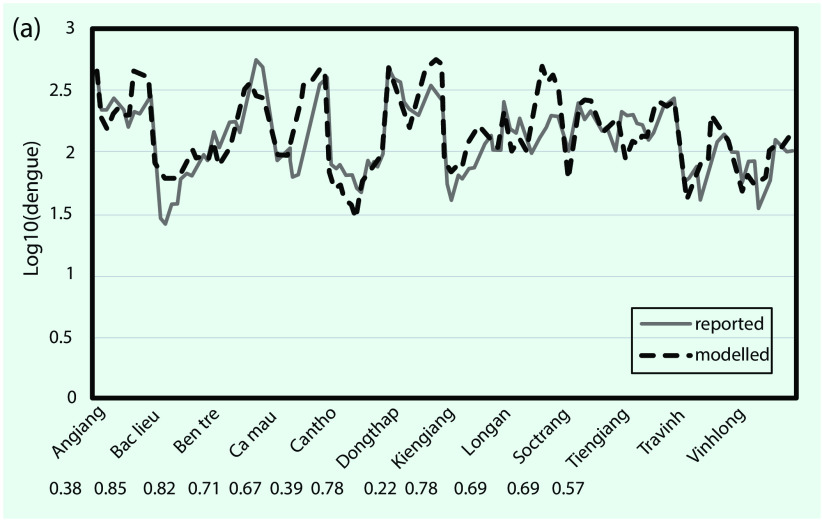
**Forcast versus reported dengue cases in log scale for whole Mekong River Delta region: (a) series plot for January–December 2016 and (b) scatter plot with regression**

[**insert** Figure 5a]

**Figure 5b:**
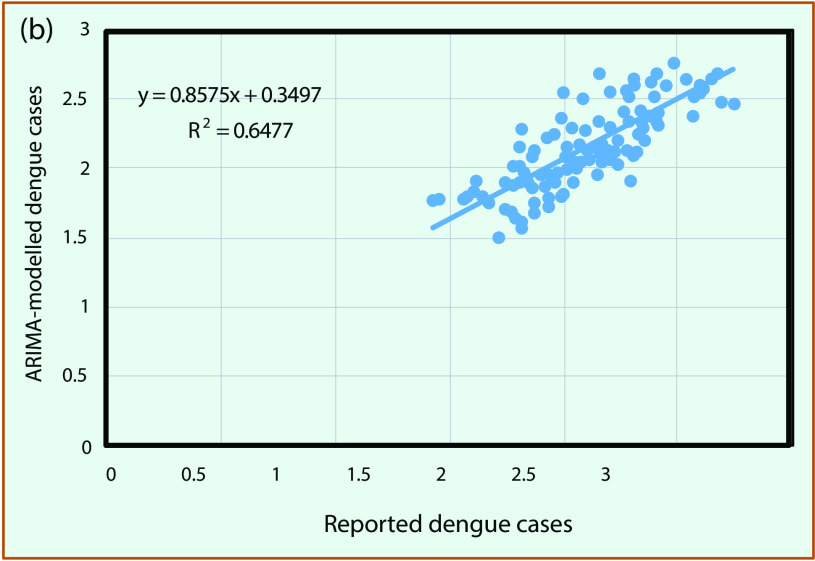
**Forcast versus reported dengue cases in log scale for whole Mekong River Delta region: (a) series plot for January–December 2016 and (b) scatter plot with regression**

[**insert** Figure 5b]

We also evaluated the association between monthly predicted and reported dengue incidence from January to December 2016 by calculating the Pearson correlation coefficient (*28*) for each province (the numbers presented correspond to the provinces in **Fig. 5a**). The correlation varies significantly, from approximately 0.22 to 0.85 (with *P* < 0.05) for different provinces; nine provinces had a correlation greater than 0.50, and only three provinces had a correlation below 0.50. We found that the predicted dengue during the period January to December 2016 was more similar to reported data for provinces in subregion II where dengue was found to have a negative correlation with NDVI and a positive correlation with LST_n_ at a 2-month time lag, as previously mentioned. For the provinces in subregion I, predicted dengue was found to have a weaker correlation with the reported data during the period January to December 2016, even though the stronger association of dengue with rain and NDVI was shown by the time-series data from the years 2000 to 2015.

## Discussion

In an effort to overcome the limitations of spatial and time scales in climate data collected from meteorological stations, we created a high-quality data set of satellite remote sensing data for climate and environmental factors, i.e. rain data from GSMaP and temperature and vegetation data from MODIS for the entire MDR for dengue modelling using a time-series approach. Different combinations of components were evaluated to construct the best predictive ARIMA models for 12 provinces across the study region. We decided to use the ARIMA model because it can cope with a stochastic dependence of consecutive data and to account for autocorrelations in time series as well as seasonality, long-term trends and time lags. (*14*) The selection of external variables was based on the best correlation of dengue with rain, LST_d_, LST_n_, and NDVI at different time lags for the 2000 to 2015 data. The results of the validation showed different performances of the ARIMA model over the region. The correlation of predicted and reported dengue during the period January to December 2016 was found higher in subregion II and lower in subregion I, controverting the distribution of dengue among these two subregions (as in **Fig. 1**). This is a limitation of ARIMA modelled with only climate factors relating to vector abundance; transmission of dengue is also affected by other factors such as population density and activities, relating human susceptibility to the disease. Therefore, an assessment of prevailing vulnerabilities to dengue could be independent of its spatial distribution, (*29*) but closely related to a combination of climate, environment and sociodemographic conditions. (*30*)

Possible reasons of inconsistent effects of NDVI on dengue incidence include subprovincial variations in precipitation and a variety of land covers. Climatologically, the onset of the rainy season in the MDR usually appears first along the coast (subregion II) and moves gradually inland (towards subregion I), resulting in a different temporal time lag in relationships between rain and dengue transmission over the region. Also, vegetation type and growth stage may play important roles in determining vector abundance, irrespective of their association with rain. (*31*) An analysis of NDVI distribution in relation to land cover data over the whole region indicated that lower values of NDVI in subregion II corresponded to more water bodies, shrubs and mixed horticulture land cover types. Higher NDVIs were found in subregion I, which corresponded to more rice paddy land cover.

Several investigators have examined the associations among climate variables, demography and dengue incidence in the southern provinces of Viet Nam using a wavelet time series analysis, (*3*, *32*) a standard multiple regression model, ARIMA and a Poisson distributed lag model. (*33*) We found that time-series analysis was useful in establishing the relationship between the change of weather parameters, environmental factors and the incidence of dengue diseases for the entire MDR. Input variables were selected based on the best correlation of dengue with precipitation, LST_d_, LST_n_, and NDVI data, for the period 2000 to 2015 at varying time lags as in previous studies. (*19*, *33*)

### Limitations

One challenge to the existing statistical approaches in modelling dengue is the difficulty of quantifying the influences of myriad human activities on vector-borne transmission. An integrated approach might be to build an index framework for underlying socioeconomic and demographic factors. (*29*) Disease transmission is also closely linked with mosquito behaviour and population dynamics that are largely influenced by climate factors. (*5*) Therefore, direct modelling by using climate data could be useful in informing health and sanitation sectors of potential increases in mosquito activity and subsequent disease risk, especially if mosquito population data can be sampled and integrated.

### Conclusions

This study focused on presenting climate and environmental factors from remote sensing data in modelling and predicting dengue fever in the MDR of Viet Nam. Our results indicate that this approach may be effective for predicting regional dengue incidence and trends. The results also revealed that the higher correlation of dengue using a single variable does not improve the model performance, and validation of the model is crucial for assessing its accuracy. Our findings support previously made conclusions that dengue prediction models vary due to their complexity and methodology and are dependent on the type of data collected and the nature of the variables. (*14*) No universal models exist for global analysis and prediction, (*14*) even if limited to a climatologically homogeneous area such as the MDR.

We describe the first effort to apply remote sensing data to perform time-series modelling of a vector-borne disease in Viet Nam to enhance the dengue early warning system. Similar approaches have been used throughout the world. (*34*-*36*) Integration of remote sensing and modelling to provide early warning of vector-borne disease outbreaks has been successfully demonstrated for malaria throughout Africa (*35*) and for dengue epidemics in Brazil. (*14*) To determine our model’s usefulness as an early warning tool, the results of our study have been presented on the Internet not only for the MDR, but also for other regions of Viet Nam and the Philippines. However, the system should be evaluated by end users for its effectiveness for dengue predictions for two countries.
